# Combining the best of two methodological worlds? Integrating Q methodology-based farmer archetypes in a quantitative model of agri-environmental scheme uptake

**DOI:** 10.1007/s10460-021-10242-w

**Published:** 2021-07-09

**Authors:** Heidi Leonhardt, Michael Braito, Reinhard Uehleke

**Affiliations:** 1 Institute for Sustainable Economic Development, University of Natural Resources and Life Sciences Vienna, Feistmantelstraße 4, 1180 Wien, Austria; 2 Institute for Food and Resource Economics, University of Bonn, Meckenheimer Allee 174, 53115 Bonn, Germany

**Keywords:** Farmer typology, Farmer archetypes, Agri-environmental schemes, Mixed methods, Q methodology, Farmer behavior

## Abstract

Increasing farmers’ acceptance and adoption of environmentally beneficial farming practices is essential for mitigating negative impacts of agriculture. To support adoption through policy, it is necessary to understand which types of farms or farmers do or do not (yet) apply such practices. However, farmers are not a homogeneous group and their behavior is subject to a complex array of structural, socioeconomic, and socio-psychological influences. Reducing this complexity, farmer typologies or archetypes are useful tools for understanding differing motivations for the uptake of sustainable farming practices. Previous investigations of the role of farmer archetypes in the adoption of such practices rely on either purely qualitative or purely quantitative methods in data collection, typology creation, and hypothesis testing. Our study combines both approaches by classifying survey respondents into farmer types based on a previous Q methodological study. We then use these types in a two-part regression model that aims to explain participation in agri-environmental schemes (AES) and the level of scheme participation. To control for farm structural factors, we additionally link our questionnaire data to secondary data from the farm accountancy data network. Results indicate that in Austria, AES are attractive to all types of farmers, but the level of participation (AES income per hectare) in these schemes differs between archetypes: Profitability-oriented farmers participate less, and nature-oriented farmers participate more than other types. This suggests that monetary compensations for sustainable farming practices are not perceived as sufficient by certain groups of farmers, and policy makers need to consider additional kinds of incentives.

## Introduction

In Europe, agri-environmental schemes (AES) are the primary policy measure to tackle agriculture's negative impacts on the natural environment. These schemes offer monetary compensation to farmers who voluntarily adopt environmentally beneficial farming practices (Ronchi et al. 2019; Zimmermann and Britz 2016). To increase AES uptake, understanding farmers’ motivations and behavior is essential. Despite the monetary compensation, changes to established farming practices often present a risk to the individual farmer. Moreover, farmers' behavior is driven by a complex interaction of agronomic, social, cultural, environmental, formal, and informal institutional determinants that vary in different contexts (Prager and Posthumus 2010; Bartkowski and Bartke 2018; Prokopy et al. 2019; Siebert et al. 2006). Therefore, understanding AES uptake or compliance requires researchers to consider farmers’ socio-psychological factors as well as farm structural and socio-economic aspects (Dessart et al. 2019; Lovejoy and Napier 1986). This, however, increases the complexity of such research.

One way of reducing this complexity is to structure all relevant aspects by defining archetypes that allow grouping farmers into a finite set of types. Each archetype captures a particular combination and peculiarity of socio-psychological, farm structural, and socio-economic aspects. Such archetypes have been used to characterize groups of farmers as well as to understand the uptake of agricultural practices (for a review see Emtage et al. 2007), including AES adoption (Guillem et al. 2012; Hammes et al. 2016; Cullen et al. 2020). However, researching archetypes comes along with methodological choices and constraints. While studies that focus on identifying and describing archetypes often use intensive and qualitative methods, studies that link archetypes to behavior tend to apply ad-hoc, quantitative data-driven methods. Both methods have obvious strengths and weaknesses: Qualitative methods allow for an in-depth understanding of farmer archetypes, but they do not produce generalizable results beyond the group of farmers under study. Quantitative methods allow investigating archetypes’ socioeconomic characteristics and their prevalence in a wider population of farmers, but this may come at the cost of theoretical depth.

Our study feeds into the existing research on farmers’ AES participation by applying archetype analysis, but combines the advantages of both approaches: We use qualitatively derived archetypes in a quantitative questionnaire survey to assess farmers’ AES participation, and combine this survey data with secondary data on farm structure and farm business performance. Since such mixed methods investigations are rare in agricultural research and elsewhere, our study provides a novel means to jointly leverage the respective strengths of qualitative and quantitative archetype research. In doing so, it addresses the following research objectives.

Our first objective and contribution to the literature is an investigation of the explanatory power of farmer archetypes when studying the determinants of farmers’ AES participation (research objective A). Similar research already exists, as archetype development is a common research tool to structure farmer heterogeneity. Archetypes have, for example, been linked to farmers’ engagement in multifunctional activities (Jongeneel et al. 2008), use of agricultural best management practices (Thompson et al. 2015), farmers’ land-use intensity and resulting on-farm biodiversity (Schmitzberger et al. 2005), adoption of low emission agricultural practices (Morgan et al. 2015), and support for soil and water protection policies (McGuire et al. 2015). These studies usually find that farmer archetypes are valuable explanatory factors of behavior, albeit results for AES adoption in particular are mixed: Hammes et al. (2016) do not find any differences in the likelihood of AES participation between different types of grassland farmers in northern Germany, Guillem et al. (2012) find some differences in past AES adoption by farmer types in Scotland, and Cullen et al. (2020) identify an impact of farmer self-identities on the likelihood of AES participation in Ireland. Our unique way of data integration allows us to gain a robust understanding of AES participation by archetypes, such that we hope to contribute to the clarification of these mixed results. This is made possible by our combination of qualitative and quantitative archetype development and its combination with secondary data, enabling us to control for farm characteristics which have been identified as relevant for scheme adoption (Zimmermann and Britz 2016). Moreover, since we will be able to identify and describe farm structural characteristics of each archetype, we can draw policy-relevant and generalizable conclusions about the relationship between archetypes and AES participation.

Our second contribution to the literature addresses AES participation itself, as we distinguish between AES participation and the level of participation in our analysis (research objective B). Most studies that focus on the role of socio-psychological factors for AES uptake compare farmers that do not participate in any scheme with those that participate in some scheme in a binary manner. However, individual schemes may differ vastly in their intensity and the required changes in production systems. Accounting for such differences requires differentiating between schemes in analysis, or separately investigating scheme uptake and the level of scheme participation in terms of, e.g., the area per farm covered by schemes or the number of schemes a farm participates in. This has been done in some studies that focus on explaining AES participation with structural and/or socioeconomic determinants (see e.g., Defrancesco et al. 2008; Giovanopoulou et al. 2011; Ma et al. 2012), but not elsewhere. Since our study design allows us to integrate primary and secondary data, we can use a farm’s AES income per hectare to distinguish between scheme participation and participation intensity and get an enriched and nuanced picture of farmers' AES uptake, again potentially explaining the mixed results of previous studies.

Methodologically, we base our work on a study by Braito et al. (2020), data from a questionnaire survey with Austrian crop farmers, as well as secondary economic data on survey respondents’ farms. Braito et al. (2020) use Q Methodology to identify farmers’ viewpoints on soil management, which can be directly interpreted as archetypes due to the nature of the method used (see following section). Since Q methodology is usually based on a small and purposely selected (not representative) sample of participants, results cannot be used to draw conclusions about the prevalence of archetypes among the farmer population or to identify relationships between archetypes and structural factors or behavioral patterns. However, Danielson (2009) presents methods for combining Q methodology with survey approaches that allow for making such links. We apply two of these methods to group survey respondents according to the viewpoints established by Braito et al. (2020), i.e. assign them to archetypes. We then link the questionnaire data to secondary data containing farm structural and economic information, including information on AES participation. This allows us to see how prevalent different archetypes are, and whether archetypes correlate with farm structural factors or AES participation. Finally, we use a two-part regression model to investigate the role of farmer archetypes for scheme uptake and the level of participation in such schemes, controlling for farm structural factors.

Overall, we hope that our study will provide a holistic and thorough example of investigating AES uptake, as it considers farm structural factors alongside farmer archetypes in a comprehensive manner, referring to primary data as well as a secondary dataset that is harmonized and collected EU-wide. Before we describe the methods and data that we employ in more detail, the following two sections review the literature on farmer typologies and introduce Q methodology as a way of establishing farmer archetypes.

## Farmer archetypes and behavior

Although research on farmer archetypes has some tradition, no unified terminology or predominant concept exists. Nevertheless, since all concepts listed below group farmers into a finite set of unique types with the goal of structuring complexity, we consider archetypes an appropriate umbrella term. The possibly most widely-known term for farmer archetypes is the concept of farming styles developed by Jan Douwe van der Ploeg (van der Ploeg 1992, 2013). However, critics claim that the concept is imprecise and misleading (Vanclay et al. 2006). In addition, semantically similar concepts exist, including farming strategies (Preissel et al. 2017), farmer types (Darnhofer et al. 2005), farming sub-cultures (Vanclay et al. 1998), farmer (self-)identities (Cullen et al. 2020; McGuire et al. 2015; Hyland et al. 2016), farming values (Maybery et al. 2005), farmers’ activity systems (van der Ploeg et al. 2009), belief systems (Thompson et al. 2015), perspectives (Walder and Kantelhardt 2018), or viewpoints (Zagata 2010; Braito et al. 2020). The understanding of what these concepts represent ranges from researchers’ mental frameworks (Vanclay et al. 2006) to actual descriptions of reality (Emtage et al. 2006), depending on the ontological stances of the researchers undertaking a study (Fairweather and Klonsky 2009). Additionally, whether such farmer archetypes should be considered mutually exclusive, or whether they overlap and farmers thus share characteristics of several types is still controversial (Fairweather and Klonsky 2009; Vanclay et al. 2006). Despite heterogeneities, all these approaches are comprehensive regarding the farm and the farmer and consider structural factors as well as farmers’ perceptions and interpretations, albeit to a varying extent (depending not only on the typology system of choice but also the data generation and classification method).

In addition to the lack of one unified concept, there is also no unified typology or ‘list’ of potential types. There has been some debate about whether a general pattern of archetypes across studies and contexts exists (Emtage et al. 2006; cf. Vanclay et al. 2006). Farmer archetypes that have been found in previous studies include – among others – business-oriented and environmentally-oriented types, types with a productivist mindset, types focused on tradition and family-farming, types focused on independence, types who are disengaged, types that farm as a hobby, and types sharing various combinations of such attributes (see for example Davies and Hodge 2007; Walder and Kantelhardt 2018; Guillem et al. 2012; Emtage et al. 2006; McGuire et al. 2015; O’Rourke et al. 2012; Hammes et al. 2016; Maybery et al. 2005). Generally, this plurality of types can be interpreted as an indicator that farmer archetypes are contingent on time and place (van der Ploeg 1992; Fairweather and Klonsky 2009).

Many of the studies mentioned thus far implicitly assume or explicitly investigate a relationship between farmer archetypes and behavior, or the outcomes of behavior (e.g., environmental outcomes). This is usually based on a framework assuming that farmer behavior is – as any other human behavior – guided by socio-psychological factors, which can in turn be captured by farmer archetypes (Emtage et al. 2007). We adopt this notion and assume that archetypes differ in their AES participation due to differences in their underlying socio-psychological characteristics. In addition, we acknowledge that other factors that are external to the farmer’s socio-psychological constitution may also influence AES adoption. These include (a) the farm’s production portfolio (e.g., farm type, farm size) and farm characteristics (e.g., geographical situation), which are important determinants of the ‘goodness of fit’ of a scheme with farm management (Zimmermann and Britz 2016); (b) farmer demographics (e.g., age, education), reflecting a capacity to change farming practices; (c) scheme characteristics (Zimmermann and Britz 2016); and (d) wider economic or societal influences external to the farm (Jongeneel et al. 2008). Since our study is confined to one country, we assume that (c) and (d) do not vary between farmers. The other factors will be controlled for in the quantitative model, enabling us to isolate the potential influence of archetypes.

Several qualitative/intensive and quantitative/extensive methods of archetype identification and creation exist. They range from qualitative interview-based methods (e.g., Darnhofer et al. 2005) to quantitative methods such as cluster analysis (e.g., Guillem et al. 2012). One method that is well-suited for identifying farmers’ archetypes in a farmer-led and (largely) qualitative fashion is Q methodology (Fairweather and Klonsky 2009; van der Ploeg and Ventura 2014). The following section describes this method in more detail.

### Q methodology as a method to identify archetypes

Q methodology has been developed as a means of understanding human subjectivity by identifying viewpoints and positions in a discourse (Watts and Stenner 2005; Zabala et al. 2018; Previte et al. 2007). It finds frequent application in socio-environmental research, including agriculture (Sneegas et al. 2021). In short, participants of a Q methodological study are asked to sort a set of statements that reflect the discourse of interest (or more generally the subject matter at hand) according to their level of agreement in a specific, often quasi-normal, shape (Watts and Stenner 2012). The resulting shapes (”Q sorts”) with the statements ranked in relation to each other are then, simply speaking, correlated to one another to identify ranking patterns that are shared by several participants. The final result of a Q study are interpretations of idealized statement rankings that represent different viewpoints in a discourse, and that are each defined and shared by a group of participants. Each viewpoint is “an archetype of those who sort in a similar way” (Fairweather and Klonsky 2009, p.191). The viewpoints are constructed in a way that minimizes overlaps between viewpoints, but some correlations may still remain. While the process of comparing and correlating participants’ sortings is a quantitative procedure, the method entails significant qualitative components in the statement ranking procedure and in the interpretation and definition of viewpoints (Watts and Stenner 2005; Zabala et al. 2018). Perhaps the most crucial step in Q methodology is the selection and development of the set of statements that are presented to participants, as this provides the frame to which answers are restricted and within which archetypes are determined. Statements thus need to be comprehensive, i.e. “*broadly representative* of the opinion domain at issue” (Watts and Stenner 2005, p.75, emphasis in original) and balanced, i.e. not biased towards one perspective (Watts and Stenner 2012).

In Q methodology, participants are grouped in terms of the viewpoint they most strongly correlate with, according to their statement sorting. Participants may also share some similarities with multiple viewpoints (although in this case they are usually precluded from *defining* a viewpoint), such that archetypes determined in this way can be considered overlapping and not completely mutually exclusive (Fairweather and Klonsky 2009). In addition, since the set of statements is comprehensive, the resulting viewpoints are also comprehensive, i.e., comprise all social and psychological aspects that are relevant. Notwithstanding this comprehensiveness, Q methodology can be geared towards a particular topic or questions, such that the resulting typology has a particular focus (or ‘point of entry’ (Vanclay et al. 2006)). Accordingly, Q methodology has not only been applied to create farmer archetypes in a general sense (Fairweather and Keating 1994; Brodt et al. 2006; Pereira et al. 2016; Zagata 2010), but also to identify farmers’ archetypical environmental perspectives (Davies and Hodge 2007, 2012; Walder and Kantelhardt 2018), attitudes towards productivity and technologies (Alexander et al. 2018), views on pesticide use (Lehrer and Sneegas 2018), views on farm succession (Barbosa, 2020), or – as in the present case – determinants of soil management (Braito et al. 2020). Choosing a particular focus may additionally aid relating resulting types to actual behavior, as (behavior-)specific attitudes are usually considered better predictors of behavior than broad and unspecific attitudes or values (Emtage et al. 2007; Ajzen 2012).

## Methods and data

As mentioned above, our archetype definition is based on a Q methodological study by Braito et al. (2020). These authors conducted their study with 33 Austrian crop farmers (selected from a range of backgrounds, e.g., with/without livestock, organic/conventional, male/female, AES/no AES, different regions) and identified four different archetypical farmer viewpoints (hereafter: archetypes) in winter 2017/18. The 34 statements (“Q set”) used by Braito et al. (2020) reflect the potential determinants of soil management: aspects relating to farm, farmer, socio-institutional context, and natural context that may determine farmers’ management choices. The statements were gathered through a literature review and six stakeholder interviews. The question to which interviewees sorted the statements on a scale ranging from -4 (disagreement) to + 4 (agreement) was “What determines how you manage your soil?”. Braito et al. (2020) identified four soil management archetypes: *Nature Participants (NP)*, driven by their relationship with nature and having a focus on innovation in soil management; *Pleasure Seekers (PS)*, sharing a focus on nature but considering personal freedom and joy as essential; *Traditional Food Providers (TFP)*, prioritizing food production and valuing traditions in managing their soil, and *Profit Maximizers (PM)*, motivated by their farms’ economic viability and profitability.

We use these four archetypes to group respondents of a questionnaire survey into four types and then model respondents’ participation in AES in an econometric model. While the archetypes primarily relate to soil management and AES also cover additional aspects, we deem it appropriate to link soil management archetypes to AES participation for two reasons: First, Braito et al.’s (2020) archetypes resemble broader archetypes from previous studies (see, e.g., Davies and Hodge 2007) and the statements used comprise almost all aspects that are relevant for the (environmental) management of a crop farm, beyond mere soil management. Farms that have a different focus (esp. grassland, permanent crops or intensive livestock production) may not be well represented by these archetypes, but our questionnaire focuses on crop farms and mixed farms only. Second, The AES relevant to such farms largely target soil management. At the time of our study, the five schemes (out of 23) that received most subsidies were all at least partly related to soil management on cropland,^1^ and among the top 10 schemes in terms of total subsidies spent there was only one that was relevant for crop farms but not soil management related^2^ (BMLFUW 2020).

### Assigning survey respondents to farmer archetypes

We transfer the Q set used by Braito et al. (2020) to our questionnaire survey in the following way (Danielson 2009). Questionnaire respondents were presented with 31 statements of the Q set and asked to indicate their agreement with each statement on a five-point Likert-type scale, ranging from “strongly agree” to “strongly disagree”. The statements were grouped into sets of 10–11 to ease respondents’ evaluation. In their German original, the first 21 statements started with the phrase “When dealing with my soil…”, which was written out only once per set to reduce the reading load for respondents. The corresponding statements were then restricted to their second half (e.g.: “…I rely on my own education and experience”). The remaining 10 statements were presented as full sentences. Compared to the original Q study, we removed three statements that had clearly been identified as consensus statements by Braito et al. (2020), i.e., statements that all archetypes had ranked similarly. Table 1 lists all statements in the order they were presented to the survey respondents; respondents’ mean responses, as well as the statements’ respective ranks (-4 to + 4) by the four archetypes.

**Table 1:** Statements from the Q set as presented to survey respondents, statement rankings by archetypes (see Braito et al. 2020), and mean survey response

ID	Statement	Q ranking by archetype				mean survey response
		NP	PM	PS	TFP	
S01	When dealing with my soil I go by the requirements and expectations of my customers	0	− 2	− 1	2	3.31
S02	When dealing with my soil I steer nature for my own use	1	1	1	− 2	4.02
S03	**Experiences of colleagues give me guidance for dealing with my soil**	0	− 1	0	**− 3**	3.44
S04	When dealing with my soil I rely on my own education and experience	1	3	2	0	4.45
S05	**When dealing with my soil I feel as a part of nature and its cycles**	**4**	0	3	− 1	4.48
S06	**When dealing with my soil I avoid doing things that would make me the subject of gossip**	**− 4**	− 3	− 3	− 2	3.17
S07	By dealing with my soil I avoid damages by natural influences (e.g., climate change, pests)	2	1	0	0	4.23
S08	**Voluntary programs and schemes are a useful guidance for how I deal with my soil, no matter whether I formally participate**	0	− 1	**− 3**	− 1	3.67
S09	How I deal with my soil ought not to have any negative impact on my neighborhood	1	1	1	− 2	4.26
S10	When dealing with my soil I work together with nature	3	2	4	2	4.67
S11	When dealing with my soil my freedom as a farmer is my main concern	− 2	− 1	2	3	3.45
S12	**My duty to provide food for society shapes how I deal with my soil**	1	0	− 2	**3**	3.91
S13	When dealing with my soil I do not think about nature	− 4	− 2	− 3	− 1	2.08
S14	**When dealing with my soil I have a responsibility for employees and helping people**	0	**− 3**	− 1	2	3.91
S15	I coordinate with my neighbors when dealing with my soil	− 3	− 4	− 4	− 2	2.88
S16	**Dealing with my soil ought to give me pleasure**	2	1	**4**	2	3.74
S17	I try new things when dealing with my soil	1	0	0	0	3.66
S18	**The economic viability of my farm is top priority for me when dealing with my soil**	− 1	**4**	0	1	3.88
S19	When dealing with my soil I think about future generations	3	2	2	1	4.46
S20	I implement expectations of society in how I deal with my soil	0	− 2	− 1	0	3.48
S21	When dealing with my soil I have a responsibility for nature	3	3	2	0	4.69
S22	When dealing with my soil I pay attention to the tidiness and neatness of my plots	− 1	2	1	4	4.21
S23	I attend training and extension services to learn more about soil use	2	2	− 1	3	4.21
S24	When dealing with my soil I avoid expensive investments	− 3	− 1	0	− 4	3.22
S25	Traditional, passed-down knowledge determines how I deal with my soil	− 1	− 1	0	3	3.58
S26	How I deal with my soil depends on agri-environmental schemes	− 2	0	− 2	− 2	2.83
S27	How I deal with my soil is determined by laws and governmental regulations and sanctions	− 2	0	− 4	− 3	3.18
S28	I would deal with my soil differently if I had more time	− 3	− 4	1	− 4	2.39
S29	The distance between a plot and my farm influences how I deal with my soil	− 1	− 3	− 2	− 1	1.99
S30	The number of years that I will still farm a plot determines how I deal with my soil	− 2	− 2	− 2	− 3	2.19
S31	The weather determines how I deal with my soil	4	4	3	1	4.42

*NP* Nature Participant, *PM* Profit Maximizer, *PS* Pleasure Seeker, *TFP* Traditional Food Provider. Statements printed in bold are used as defining statements in the scale creation method

We apply two different methods to group our survey respondents according to the archetypes: the “scale creation method” (SC method) (Danielson, 2009; also presented by Brown (2002) and Baker et al. (2010) as “standardized factor index score”) as well as the “profile correlation method” (PC method) (Danielson 2009). To avoid confusion between the archetypes as identified by Braito et al. (2020) and the individual survey respondents (partly) sharing these archetypical views, we will refer to the latter as a farmer’s “type” hereafter.

For the SC method, we select two defining statements for each archetype. These selected statements need to fulfill two criteria (Danielson 2009): salience (i.e., the respective archetype agreed or disagreed strongly with these statements) and distinction (i.e., the respective archetype differed (significantly) from other archetypes in its agreement with these statements). Whether a statement is ‘distinguishing’ for one archetype to satisfy the latter criterion can be determined by statistical significance (this is also used in Q methodology itself). However, in some cases, the statistically-determined “distinguishing statements” for an archetype in Braito et al. (2020) do not satisfy the salience criterion. In these cases, we select statements that are salient and clearly representative of the respective archetype in a more qualitative sense. For example, we choose the statement “managing my soil ought to give me pleasure” as a defining statement for the *Pleasure Seeker* archetype because it is at the core of the archetype, even if it only weakly distinguishes the archetype from others. In Table 1, all defining statements are printed in bold.

After determining these defining statements, we create a score for each survey participant on each archetype. Table 2 illustrates this process by means of an example; participant 58, who is defined as a *Nature Participant* type according to his/her responses and the resulting maximum (normalized) viewpoint score. This process involves the following steps: 1) reverse code participant responses (PR) to those statements that the archetypes placed on the negative side of the Q distribution, creating PR’, 2) multiply PR’ with the absolute value of this statements’ archetype ranking (AR) to create the participant score (PS) for each statement, 3) sum the PS values per archetype to obtain an archetype score (AS), and 4) normalize the AS into T-scores (mean: 50, standard deviation: 10) to account for differences in the attainable maximum scores. We then assign to each participant the type that she/he scores highest on.

**Table 2:** Example for determining one respondents' type based on the scale creation method

Statement	Defining for	Archetype ranking (AR)	Participant response (PR)	Reverse coded (PR’)^a^	Participant score (PS)^b^	Archetype score (AS)^c^	Archetype T-score^d^
S3	TFP	− 3	2	4	12	24	61.74
S12		+ 3	4	4	12		
S5	NP	+ 4	5	5	20	40	68.62
S6		− 4	1	5	20		
S14	PM	− 3	5	1	3	19	43.80
S18		+ 4	4	4	16		
S8	PS	− 3	5	1	3	23	52.07
S16		+ 4	5	5	20		

^a^if AR < 0: PR’ = 6-PR; else PR’ = PR

^b^PS =|AR| * PR’

^c^AS = ∑PS by archetype

^d^normalized AS

For the PC method, Danielson (2009) again suggests presenting a number of representative statements per archetype to survey respondents and then correlate each participant’s responses with the rankings of these statements by each archetype. Compared to the SC method, each archetype needs to be represented by a larger number of statements to allow for meaningful correlation results. These statements do not (all) need to be salient but can also be located in the middle of the Q distribution. We utilize all 31 statements presented to survey respondents. This slightly modified version of Danielson’s method (he suggests selecting only a subset of statements) reduces the subjective judgment required for selecting representative statements. Aside from this modification, we proceed as suggested and correlate each participant’s responses with each archetype’s Q rankings, using a Spearman correlation. In essence, we correlate each row of our dataset with one row per archetype that contains this archetype’s ranking. This produces correlation scores for each survey respondent with each archetype that we directly use in our further analysis. Therefore, an individual participant may correlate positively with each or multiple of the four archetypes to some degree.

### The econometric model

The farmer types determined by the SC and the PC methods then serve as our explanatory variables of interest in econometric models of AES participation. AES participation consists of two decisions that we can model conjointly or separately: a farmers’ decision to participate in any AES at all, and a farmers’ decision on the level of participation in AES; i.e., the decision on the number of schemes to participate in or the intensity of these schemes (e.g., schemes that require substantial changes to the farming operation vs. schemes that require little change). We define both decisions in terms of (the existence of) per-hectare AES income. As non-participants have zero AES income the dependent variable is censored at zero.

Depending on theoretical and statistical considerations, several modeling options for zero-censored dependent variables exist (for helpful discussions of these options, see for example Madden (2008), Humphreys (2010), and Carlevaro et al. (2009)). Our model choice is based on the following considerations. First, we consider all zeros as true zeros that arise from one mechanism: non-participation as a matter of principle (as opposed to, e.g., non-participation due to AES payments being too low). This appears reasonable, given Austria’s ‘broad and shallow’ approach to AES that results in very easy access to several low-level schemes for all potentially interested farmers (the Austrian agri-environmental program explicitly aims at achieving comprehensive AES coverage of all agricultural land). Second, we wish to investigate actual (not potential) outcomes, and to consider the participation and level of participation outcomes separately, since we suspect that farmer types may play different roles in the corresponding decisions. This leads us to the use of a two-part model, which essentially consists of a Probit model to model participation, combined with an OLS regression model of the level of participation for participants only (Belotti et al. 2015; Madden 2008).

The Probit model (first part of the two-part model) is used to estimate the probability of a positive outcome $Y$, i.e., an AES income above zero, *ϕ* (Y > 0) = Pr(*Y* > 0 | ***X***, ***T***), where $\boldsymbol{T}$ is either a set of dummies representing survey respondents’ farmer types based on the SC method or the set of correlation coefficients for each type based on the PC method, and $\boldsymbol{X}$ is a vector of control variables (see below). To model the participation level decision in the second part, we model *ϕ* (Y|Y > 0, ***X***, ***T***), again using the same $\boldsymbol{T}$ and $\boldsymbol{X}$ as above, in an OLS regression specified as $\text{for\;all}\;Y|Y\; > \;0,\;Y\; = \;\alpha \; + \;{\varvec{\beta T}}\; + \;{\varvec{\gamma X}}\; + \;\varepsilon,$

where $\alpha$ is an intercept; $\varepsilon$ is the error term; and $\boldsymbol{\beta }$ and $\boldsymbol{\gamma }$ are vectors of parameters to be estimated.

For comparison, we also estimate a linear OLS regression model where we treat the two decisions (participation and participation level) as one. All calculations were done in R (R Core Team 2018).

As outlined in the introduction, we expect that a farm’s production portfolio and characteristics of the farm(er) are related to AES participation (Arata and Sckokai 2016; Pufahl and Weiss 2009; Zimmermann and Britz 2016). We therefore include the following control variables $\boldsymbol{X}$ in all models: the log of farm size (utilized agricultural area (UAA) in ha), cattle density and the density of pigs and poultry (both in livestock units (LU) per ha), the farms’ rental share (share of rented UAA), productivity (all outputs/all inputs), whether the farm receives any payments for being situated in a least favored area (LFA, dummy variable), whether the farmer has finished education of ‘Matura’ (graduation exam from secondary school, permitting university entrance) or higher (dummy variable), and the farmer’s age (in years).

### Data and variables

The implementation of our model draws on two main data sources: Austrian data from the EU’s farm accountancy data network (FADN), and a survey with Austrian farmers that participate in the FADN. The FADN collects annual harmonized micro-economic data on commercial farms in all EU countries to evaluate their income and the impact of the Common Agricultural Policy (CAP). Data are gathered via stratified samples by national agencies. While aggregated data are freely available online, these agencies (in Austria the Federal Ministry of Agriculture, Regions and Tourism (BMLFUW)) provide farm-level data to scientists for research purposes. We use these farm-level data as control variables on farm structure and economic indicators and for our dependent variable on AES income.

In Austria, a vast majority of farmers participate in AES. Correspondingly, only 19 (6.6%) of the farmers in our sample have an AES income of zero (“zero participants”). This is partly due to the existence of a scheme that has farming requirements almost identical to the Austrian ‘greening’ requirements for the CAP’s first-pillar payments (BMLFUW 2015) and that is therefore accessible to almost all farms with little additional effort. To account for this, we subtract the potential payments for this most basic scheme, ‘environmentally sound and biodiversity-promoting management’, from the total sum of payments. This corresponds to approximately 45€ per ha, depending on total UAA and type of farmland. In our case, AES participants are therefore defined as farmers who participate in more than just this basic scheme, raising the number of non-participants in our sample to 40 (13.9%). In terms of summary statistics, the group of ‘non participants’ does not differ fundamentally from the group of ‘zero participants’: both have a UAA that is significantly smaller, and an average number of pig/poultry LU per ha that is significantly higher than the total sample.

To determine farmers’ types (as described above) and to include information on respondents’ age and education level in our model we use the data collected in an online questionnaire survey. The survey was conducted in spring 2018 and was sent out to the 1,147 FADN farmers (out of a total of 1,879 FADN farms) who farmed at least 5 ha of cropland and rented part of this land. The survey consisted of three sections: the first section was part of a study on agricultural land renting (not used here), a second section contained the Q statements presented in Table 1, and a third section asked for additional sociodemographic information (only information not included in the FADN data). To connect the survey data to the economic FADN data, respondents were required to enter their FADN farm ID at the beginning of the survey. The entire questionnaire took about 20–30 min to complete, with the first section being the most time-consuming. No debriefing questions for reliability checks were included; however, we did not see any obviously unreliable results (such as identical responses to an entire block of statements). The tax and accountancy consultancy firm that administers the FADN data collection on behalf of the Austrian federal ministry pre-tested the questionnaire, identified and contacted farmers, sent out the survey invitations, and encouraged farmers to participate via e-mail, phone calls, and during their annual farm visits.

We attained a response rate of 31% with 344 fully completed questionnaires. Considering that the survey was a lengthy and voluntary online survey with no incentives or compensation attached, this is a reasonable response rate for a social science study (Sauermann and Roach 2013), and is comparable to other farmer studies (Avemegah et al. 2020). Since contact details of the farmers remained with the consultancy managing the questionnaire, we did not conduct non-response bias checks. However, since we have FADN data for all farms that were contacted for the survey, we can compare respondents and non-respondents to some extent (see below). A total of 300 respondents provided a correct FADN ID, enabling us to use their data for our analysis. We further excluded permanent crop farms and farms with an output share of > 49% of vegetables from our analyses, since their structure and AES income differs considerably from other farms. We additionally excluded one farm with very high leverage from the model, as we could not determine why that farm had an unusually high AES income.

Table 3 compares FADN data for respondents and eligible non-respondents to evaluate the representability of the sample. It shows that significant differences exist with respect to farm size, cattle density, and rental share. All these differences are likely a result of the study focus on farms with cropland and rented land. Farms with cattle and with little rented land are therefore underrepresented and results may not be fully transferrable to such farms. In the outcome variable – AES payments per ha – respondents do not differ significantly from non-respondents.

**Table 3:** Comparison of survey participants and nonparticipants (arithmetic means)

Variables	Survey respondents^a^	Non-respondents	Significance of difference in median
Number of farms	288	863	
AES payments (€/ha)	166.13	160.55	–
UAA (ha)	52.75	46.29	**
Cattle density (LU/ha)	0.45	0.65	***
Pigs/poultry density (LU/ha)	0.81	0.61	–
Rental share (of UAA)	0.46	0.40	***
Productivity (Inputs/Outputs)	1.13	1.16	–
LFA payments (€/ha)	42.33	50.96	–

***p < 0.001, **p < 0.01, *p < 0.05. Test for significance in differences: Wilcoxon rank-sum test for non-normally distributed data

^a^ includes only those in the final sample (correct FADN ID, not excluded for farm type)

For the 288 survey respondents, Table 4 shows descriptive statistics of the variables used in the model; for the full sample and by farmer type as determined by the SC method. For variables taken/computed from the FADN, Table 4 also provides the variable names as defined and used by the European Commission (2020).

**Table 4:** Summary of the variables used in the regression model (arithmetic means and percentages), including FADN variable names

Variables	Full sample	SC type NP	SC type PM	SC type PS	SC type TFP	FADN variable name(s)
Number of farms	288	75	82	64	67	
Number of non-participants	40	10	13	12	5	
AES payments (€/ha)^a^	126.56	144.21	97.86	117.48	150.62	SE621 / SE025
UAA (ha)	52.75	43.76	58.22	42.23	66.20	SE025
Farms with cattle (%)	39.93	46.67	37.80	45.31	29.85	SE085 + SE090 > 0
Cattle per ha if any (LU)	1.14	1.09	1.25	1.25	0.89	SE085 + SE090 / SE025
Farms with pigs/poultry (%)	46.18	50.67	40.24	60.94	34.33	SE100 + SE105 > 0
Pigs/poultry per ha if any (LU)	1.75	1.18	2.30	1.53	2.27	SE100 + SE105 / SE025
Rental share	0.46	0.43	0.47	0.46	0.49	SE030 / SE025
Productivity (Outputs/Inputs)	1.13	1.14	1.14	1.09	1.14	SE132
LFA (%)	58.33	65.33	52.44	70.77	46.27	SE622 > 0
Higher education (%)	21.53	26.67	20.73	15.38	22.39	
Age	49.12	48.55	48.74	47.83	51.45	
PC type NP (mean correlation)	0.50	0.60	0.45	0.45	0.51	
PC type PM	0.48	0.50	0.51	0.43	0.46	
PC type PS	0.40	0.45	0.38	0.44	0.33	
PC type TFP	0.30	0.31	0.22	0.28	0.38	

^a^Above potential AES income from basic scheme

*SC type* type as determined by scale creation method, *PC type* by profile correlation method

*NP* Nature Participant, *PM* Profit Maximizer, *PS* Pleasure Seeker, *TFP* Traditional Food Provider, *UAA* utilized agricultural area, *LU* livestock unit, *LFA* least favored area

One observation in Table 4 worth mentioning concerns the prevalence of and relationship between the four farmer types defined by the two different methods. The first row of the Table shows the number of farms per type in our sample (as defined via the SC method). Here, the different farmer types appear to be distributed rather evenly among the general survey population. As the second row shows, this is also true for the AES non-participants, with the exception of the TFP type. The bottom four lines of the Table show the mean correlation coefficients of respondents with the archetypes as calculated by the PC method. Here we see that overall the correlation with archetypes varies, and survey respondents’ mean correlation with the *Traditional Food Provider* and *Pleasure Seeker* archetypes is lower than with the other archetypes (correlation scores for non-participants – not shown here – are very similar). The Table also shows that the different ways of identifying types do not lead to identical results, as PC correlation scores with one archetype are not necessarily highest for those assigned to the same type according to the SC method.

From Table 4 it also becomes evident that some substantial differences between types exist concerning AES payments, but also concerning other farm characteristics such as UAA and the presence of livestock. Therefore, it is essential to include farm structural variables as controls in our analysis of the relationship between farmer types and AES participation.

## Results

Table 5 presents the results for the farmer types as determined by the SC method, for the two-part model and the full sample OLS for comparison (column 2). The different types are included as a set of dummies, where the *Profit Maximizer* type serves as the baseline comparison. In the two-part model, the average marginal effects (AME, column 3) and OLS coefficients (column 4) for the other types (rows shaded in grey) show no significant differences in the likelihood of AES participation, but some differences in the level of AES participation between types. In particular, *Profit Maximizer* type farmers participate at a lower level in AES than others, especially compared to *Traditional Food Provider* and *Nature Participant* types, although the latter result is only significant at the 10% level. Considering the magnitude of the coefficients, a *Traditional Food Provider*-type farmer who is an AES participant has, on average, an AES income that is 36€ per ha higher than the AES income of an identical *Profit Maximizer*-type farmer (95% confidence intervals: 2.84 – 69.65). Given that the average AES income for participants is 180€ per ha, this is quite a substantial difference.

**Table 5:**
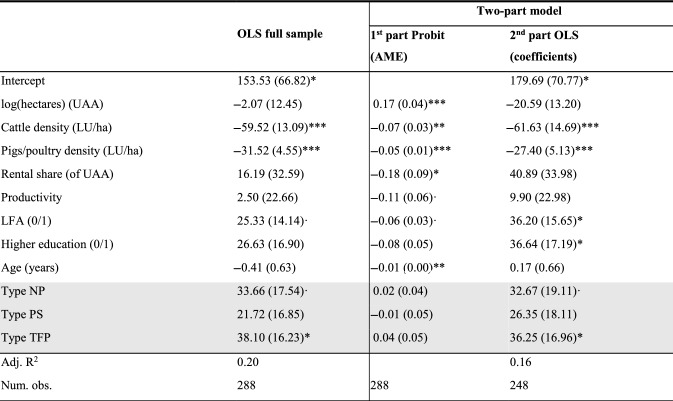
Model results for AES participation by farmer types as determined by the scale creation method

***p < 0.001, **p < 0.01, *p < 0.05, ·p < 0.1. Robust standard errors in parentheses

*AME* average marginal effects, *NP* Nature Participant, *PM* Profit Maximizer, *PS* Pleasure Seeker, *TFP* Traditional Food Provider, Base effect: Farmer type PM, LFA = 0, higher education = 0

Table 6 presents the same model results but for the farmer types as defined by the PC method. Here, correlation coefficients of participants with each archetype represent the types. In general, the results resemble the ones from the SC method, but they are more pronounced in terms of statistical significance. In the first part (Probit) of the two-part model, we find that resembling the *Pleasure Seeker* archetype is negatively correlated with the likelihood of AES participation (third column). In the second part (fourth column), we see that resemblance with the *Nature Participant* archetype correlates positively (and significantly) with the level of AES participation, and the opposite is true for the *Profit Maximizer* archetype. Interestingly, and in contrast to the findings presented in Table 5, we do not find any relationship between the *Traditional Food Producer* archetype and AES participation level.

**Table 6:**
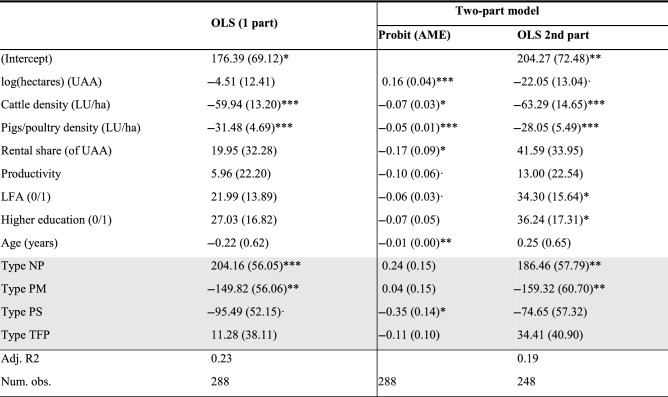
Model results for AES participation by farmer types as determined by the profile correlation method

^***^p < 0.001, **p < 0.01, *p < 0.05, ·p < 0.1. Robust standard errors in parentheses

*AME* average marginal effects. *NP* Nature Participant, *PM* Profit Maximizer, *PS* Pleasure Seeker, *TFP* Traditional Food Provider. Base effect *LFA* 0, *higher education* 0

Therefore, both methods of identifying farmer types show that AES participation levels partly depend on farmer types. This is less true for participation as such. Moreover, while overall results are similar, some differences between the SC and PC methods exist.

Concerning control variables, we see in all models that the more livestock intense a farm is, the less likely it is to participate in AES and the lower its participation level. Farms with more UAA, a lower rental share, and a younger farm manager are associated with a higher likelihood of participation. Being situated in an LFA and having a high educational level are associated with a higher participation level. The difference in influential variables between the two decisions indicates that a two-part model has additional explanatory power compared to models that combine participation and the level of participation in a single model.

## Discussion

### Farmer types and AES participation

Overall, our results suggest that classifying farmers into archetypes provides explanatory value when modeling behavior such as AES participation. In particular, farmer types that in our case reflect farmers’ viewpoints about soil management appear to be related to farmers’ level of participation in AES, but not to participation: Different types are equally likely to participate in at least one scheme, but exhibit different levels of participation. This adds to the literature investigating the determinants of AES participation and underlines the importance of accounting for farmers’ social and psychological aspects in such studies and in studies evaluating AES outcomes.

Let us consider the results for each farmer archetype in turn. The results for *Nature Participants* and *Profit Maximizers* are rather consistent across models and type specifications. *Nature Participant* type farmers tend to show a higher level of AES participation than others, which is statistically more significant in the PC method case, but also visible using the SC method. This result is not surprising and reflects this archetype’s definition as being driven by environmental concerns and placing great importance on their relationship with nature. It also shows that farmers of this type, who have an intrinsic motivation to apply environmentally friendly farming practices, use the opportunity to receive subsidies for doing so. The results for the *Profit Maximizer* archetype are somewhat more surprising. These farmers participate in AES at a lower level than other types, although AES payment levels are supposed to compensate farmers for any losses that occur due to the change in their farming operation, and have sometimes even been criticized as overcompensating farmers (Mennig and Sauer 2020). Therefore, scheme participation should – in theory – have little or no economic impact on a farm (or even a positive impact, if payment levels are indeed higher than the costs incurred). Moreover, while the *Profit Maximizer* archetype is primarily motivated by its farming operation’s economic viability, it still shares some concern for nature (Braito et al. 2020) and should thus have at least some interest in applying pro-environmental practices. Our results, however, suggest that *Profit Maximizer* type farmers do not perceive the practices that intense schemes require as profitable for their farming operation, i.e., as not providing enough (perceived) compensation for (perceived) potential losses. These farmers thus appear to require additional incentives for participating in higher level AES.

The results for the other two types are less pronounced and less consistent across models and type specifications and should therefore be considered with some caution. Farmers of the *Pleasure Seeker* type are somewhat less likely than others to participate in AES. At first sight, this is surprising since Braito et al. (2020) define the corresponding viewpoint as ecocentric and show that concerns for nature are important for these farmers’ soil management. However, this archetype is also defined by a focus on freedom, which may be understood as freedom from rules and bureaucracy. Therefore, while this type considers nature and the environment to be important for soil management, this may not be reflected in AES participation due to other counteracting mechanisms such as a desire to avoid paperwork or constraints in decision-making. *Traditional Food Provider* types may, from the outset, be expected to be less likely to participate in AES than others, as AES usually aim at supporting the provision of environmental amenities instead of food and feed. If *Traditional Food Provider* type farmers value food production more than other things, they should thus be less likely to apply farming practices that have environmental effects at the possible expense of food production. Although rather weak, our results indicate the opposite: *Traditional Food Provider* type farmers participate in AES at a higher level than others and in particular compared to *Profit Maximizer* type farmers. They also have the highest mean AES income of all groups. We can only speculate that such farmers perceive food production and AES participation as not mutually exclusive.

Braito et al. (2020) provide suggestions about how to address all archetypes through policy if one wishes to encourage more environmentally friendly farming practices. They stress that AES should be complemented with other policy options in order to be inclusive. However, some of their suggestions can also be applied to AES design and framing. For example, addressing specific types of human-nature relationships (Flint et al. 2013; Braito et al. 2017) appeals to all farmer types, such that framing policy options in this way may increase their attractiveness. In addition, Braito et al. (2020) suggest that addressing Nature Participants and Pleasure Seekers via social networks may be a successful option. In the AES context, this could be realized through collaborative AES. Whatever the policy choice, our approach has the invaluable benefit that we can identify demographic and farm structural characteristics of different farmer types. This makes it easier for extension agents to identify and target particular types of farmers and adjust their way and focus of communication accordingly. In addition, this enables policy makers to tailor schemes better to particular farm(er) types. The above average livestock density of *Profit Maximizer* types, for example, suggests that this may be a factor in the (non-)uptake of higher level schemes and therefore a point to consider in scheme design.

### Context and transferability

When drawing conclusions about AES participation, the specificities of the Austrian situation need to be considered. Austria explicitly aims to reach comprehensive coverage of as much farmland as possible, which is reflected in the design of AES. Several low-level schemes aim to give farmers an incentive to follow at least a minimum of environmental protection, and higher-level schemes exist for those who are willing to do more. Overall, both payment as well as participation levels are among the highest in the EU (Zimmermann and Britz 2016) and support for environmentally beneficial farming practices, including organic farming, is high among producers and consumers. Results may therefore be transferrable to countries with similar conditions (such as Sweden, Finland and Slovenia (Zimmermann and Britz 2016)), but not to countries that follow a ‘deep and narrow’ strategy in AES design (focused schemes for targeted areas). It appears plausible that in countries similar to Austria, farmer archetypes are related to the *level* of participation rather than participation per se, while in ‘deep and narrow’ AES countries farmer archetypes may be related to *participation* rather than participation levels. The number of “zero participants” in our study (those 19 farmers that participated not even in the minimum scheme) does not allow for any statistical conclusions, but gives an indication that true zero participation may, in larger samples (i.e., countries with fewer participants), be related to farmer types: in this group, the prevalence of farmer types differ from the larger ‘non participant’ group, with NP and TFP farmers being under-, and PS and PM farmers being overrepresented (although this is only true for the SC method). One interesting direction for future studies would therefore be a comparison of different countries to investigate whether the relationship between farmer archetypes and AES participation (levels) depends on country specific design of AES.

In addition, transferring farmer archetypes to other contexts may be possible to some extent (since parallels to archetypes identified in other studies and contexts exist), but conclusions about their prevalence should not be made. We suspect that Austria’s long tradition in policy support for environmentally beneficial farming or its particular agricultural structure may have an impact on the incidence of particular farmer archetypes. For example, much of the country’s area is not suited for maximizing production, such that TFP types may be less prevalent in Austria than in other countries. In a similar vein, readers should be cautious not to draw any conclusions about causality in our study. Participation in AES may induce learning about the environment as well as about the environmental effects of certain farming practices and may thus change farmers’ thinking about and understanding of their work. Therefore, past AES participation may impact a farmer’s mindset and thus their assignment to a particular archetype. Our findings do not account for this, but for correlations at the time of the study only.

### Methodological considerations

Our study shows that building farmer archetypes based on a Q methodological study and combining it with a survey and secondary data on actual behavior can provide valuable insights by its combination of qualitative and quantitative parts. Since viewpoints determined by Q methodology contain multiple socio-psychological dimensions at once, the corresponding archetypes are more multi-faceted than any single behavioral determinant—such as attitudes or preferences—alone. This makes their interpretation more challenging, but also means that the resulting farmer types reflect a farmer’s personality in a holistic way. At the same time, the approach allows the researcher to be specific with respect to the object of the study; in our case the focus was on soil management instead of, e.g., the approach to farming as a whole. This specificity may increase the explanatory power of the archetypes. Since AES for crop farms largely target soil management and conservation, we deem this approach appropriate for our present case. However, in any such study, the breadth of focus needs to be considered on a case-by-case basis to ensure a match between the specific archetypes and the behavior of interest. Overall, we can clearly recommend our methodological approach for future studies that aim to investigate quantitative questions related to archetypes; in agriculture and elsewhere.

Nevertheless, we also want to point out some important differences between the Q study and the questionnaire instrument that need to be considered. First, in a Q study all statements are evaluated and positioned in *relation* to one another, while in a questionnaire each statement is usually evaluated *individually* on a Likert-type scale. These are clearly two different approaches (Baker et al. 2010) that may potentially lead to different outcomes. Despite the differences in data generation, our survey responses appear similar to the archetype statement rankings. (Consider, for example, all statements that have a mean survey response below the middle point of the Likert-type scale in Table 1. These are all statements that have been ranked negatively by all archetypes.) Still, consistency across types and statements between both approaches is not guaranteed. Future research should investigate how large such deviations might be. However, the difference between individual and relative evaluation of statements not only a bug, but also a feature: it makes the evaluation task much easier and less complex for survey respondents.

Second, the selection of only a subset of distinguishing statements for identifying types in the SC method means that type identification from the questionnaire will be based on less information than in the original Q study. Brown (2002, p.120) contends that distinguishing statements indeed “stand out as critical markers whose patterned relationships are such as to give definition to the four viewpoints”, justifying their use for such an endeavor. However, things may not always be as clear as suggested by Brown (2002), especially if distinguishing statements are not salient (Danielson 2009), which was also the case in our study. Identifying types via the PC method may be a better choice in this case, as they consider more statements and thus aspects of farmer archetypes than the SC method. The corresponding strength of the SC method is, however, that it allows for shorter survey with fewer statements.

Third, assigning respondents to a single type when applying the SC method “may … contradict some of the basic ideas behind Q”, as Baker et al. (2010) note. In a conventional Q methodological study, some participants identify and correlate strongly with only one viewpoint, while others correlate with more than one viewpoint at once and are somewhere ‘in-between’ viewpoints. The SC method does not account for this, but the correlation coefficients produced by the PC method do, capturing overlaps. However, especially when the research interest lies in gathering demographic information about farmer archetypes, the SC method and its separation into marked-off groups allows for more straightforward conclusions. This may be particularly useful for policy-related questions, such as when extension services wish to target particular groups of farmers.

As becomes clear from these points, both the SC and PC methods have their advantages and drawbacks, and the choice of method should be made depending on the research context. To guide such decisions, the discussions of the two methods presented here as well as the discussions and additional methods proposed by Danielson (2009) and Baker et al. (2010) are helpful starting points.

Last, turning to the regression model, modeling both the participation decision and the decision about the participation level separately has proven to be helpful for understanding behavioral drivers, allowing for differentiated results. AES are generally attractive for farmers of all types, but more intense schemes or combinations of multiple schemes are less attractive to some farmer archetypes than to others. Given that the extra effort for running such a two-part model instead of a one-part model is small, we recommend this approach. The potential additional insights are valuable; policymakers, for example, may use them to inform future scheme design or scheme promotion. As an example, increasing participation in environmentally beneficial farming practices in Austria appears to require a promotion of or change in the design of higher level schemes. Low-level schemes are already accepted broadly and by all groups of farmers, while higher level schemes are not attractive to farmers that prioritize profitability. To convince these farmers, schemes may require more monetary compensation or their potential (long-term) economic benefits need to be emphasized more.

## Summary and conclusions

In this study, we apply an innovative method for creating farmer archetypes based on farmers’ viewpoints on soil management. We use the results of a preceding Q methodological study in a survey and combine the survey data with secondary farm-level economic data. We investigate whether farmer archetypes differ in their uptake and level of participation in AES. We find that in Austria, AES are generally attractive to all types of farmers. However, the participation *level* in these schemes differs between types: Profitability-oriented farmers participate in AES at a lower level than other types, and nature-oriented farmers participate at a higher level.

These results suggest that it is essential to consider socio-psychological factors, e.g., by means of creating farmer archetypes, when investigating AES participation. We show that combining Q methodology and a survey in a mixed-methods design offers a comprehensive and promising way of doing so. Such an approach not only provides sound and valid results for future research to build upon, but also increases the usefulness of archetype research for policy making.

## Abbreviations

AES: Agri-environmental scheme
AME: Average marginal effects
BMLFUW: Austrian Federal Ministry of Agriculture, Regions and Tourism
CAP: Common Agricultural Policy (of the EU)
EU: European Union
FADN: Farm Accountancy Data Network
LFA: Least favored area
LU: Livestock unit
NP: Nature Participant
OLS: Ordinary least squares
PC method: Profile correlation method
PM: Profit Maximizer
PS: Pleasure Seeker
SC method: Scale creation method
TFP: Traditional Food Provider
UAA: Utilized agricultural area

## Footnotes

1 These schemes were (in descending order of subsidies spent): “organic farming”, “environmentally sound and biodiversity-promoting management”, “greening of arable land – intercropping”, “nature conservation”, and “preventative groundwater protection” (for a description of schemes see BMLFUW (2015), for subsidy data see http://www.agraroekonomik.at/index.php?id=gruenerbericht).

2 The scheme “renouncement of fungicides and growth regulators in cereals”.
